# A Twist in the Tale: Rare Diagnosis of Lipomatosis of the Appendix

**DOI:** 10.1155/cris/5436727

**Published:** 2026-09-29

**Authors:** David Aini, Nicholas Markovic, Emily Rienzo, Arthur Topilow, Glenn Parker

**Affiliations:** ^1^ Research Division, Jersey Shore University Medical Center, Neptune Township 07753, New Jersey, USA, jerseyshoreuniversitymedicalcenter.com; ^2^ Department of Surgery, Jersey Shore University Medical Center, Neptune Township 07753, New Jersey, USA, jerseyshoreuniversitymedicalcenter.com

## Abstract

A 66‐year‐old white male with a history of diverticulosis and prediabetes presented with right lower quadrant (RLQ) abdominal pain lasting 1 month. On physical examination, the abdomen was soft without tenderness or palpable masses. CT imaging showed an enlarged appendix with a diameter of 1 cm with no surrounding inflammation. A laparoscopic appendectomy was performed, and the patient’s symptoms resolved within 3 weeks. Histopathology revealed lipomatosis of the appendix, an extraordinarily rare diagnosis which has only been reported five times prior to our case. This case study aims to increase available information on this disease to aid future diagnosis and treatment.

## 1. Introduction and Background

Lipomatosis is a benign, diffuse proliferation of adipocytes characterized by lipid hyperplasia and hypertrophy and without lipodystrophy [1]. It is often correlated with elevated BMI and insulin resistance. Insulin receptors activate lipogenesis through the IRS‐mTOR‐PKA pathway, causing abnormal growth of adipocytes [1]. With insulin resistance, many tissues are unable to process physiological levels of insulin, causing hyperinsulinemia; consequently, organs like the liver, which still process insulin, produce excessive lipocyte growth [2]. These lipocytes can then spill over into other parts of the body.

Most lipomatoses arise from white adipose tissue (WAT), of which 90% is stored in nonvisceral organs. There are other lipomatoses, like hibernomas, that arise from brown adipose tissue (BAT), but they are less common [3]. Visceral lipocyte growth differs by sex: women are less prone to hypertrophy in the omentum because they have smaller deposits, influenced by hormones like estrogen and progesterone [3].

Lipomatoses are generally asymptomatic and nonpainful but may require surgical intervention on a symptomatic basis [1]. Weight loss can alleviate symptoms, but standard techniques are not always successful due to tissue fibrosis [1]. Treatment options vary for specific types of lipomatoses but include liposuction, lipectomy, and drugs like tricyclic antidepressants or analgesics [1]. Here, we present a man found to have an extremely rare case of lipomatosis in the appendix.

## 2. Case Presentation

A 66‐year‐old Caucasian male with a history of diverticulosis, prediabetes, hyperlipidemia, hypertension, gastroesophageal disease (GERD), and a cerebrovascular accident (2019) presented with 1 month of right lower quadrant (RLQ) pain. It was described as sharp with a 5/10 intensity rating, but the symptoms subsided before his visit the following month.

Upon physical examination, the abdomen was soft, without tenderness or masses. Vital signs were stable (BP 130/82 mmHg and HR 82 bpm), and the patient was in no visible distress. The white blood cell count was within normal limits at 5.9 × 10^3^/µL, with a normal neutrophil percentage of 68.7%. The patient was overweight with a BMI of 28.37 kg/m^2^, and blood tests showed very slight hyperglycemia at 112 mg/dL.

Oral and IV contrast‐enhanced CT imaging of the abdomen/pelvis displayed a thickened appendix, measuring about 1 cm in diameter (Figure 1) (>0.6 cm is considered abnormal) [4]. No appendiceal wall enhancement, appendicolith, or periappendiceal fat stranding was seen.

**Figure 1:**
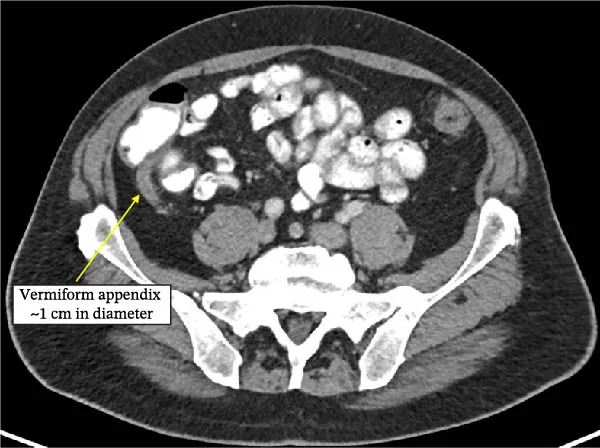
CT scan of abdomen and pelvis acquired following intravenous administration of 100 mL of Optiray (loversol), 350 mg/mL, and oral contrast (Readi‐Cat 2 Barium Sulfate). The vermiform appendix can be seen next to the cecum and measures around 1 cm in diameter.

The patient was referred to a colorectal surgeon for a laparoscopic appendectomy. The trocars were placed supraumbilically, suprapubically, and at the left midclavicular line. The appendix was divided at its base, and the abdomen was irrigated. Postoperatively amlodipine, irbesartan, aspirin, hydrochlorothiazide, and rosuvastatin were given for treatment of hypertension and hyperlipidemia. Three weeks postoperative, the patient reported no pain or complications.

Gross pathology reported an intact appendix, measuring 7.2 × 1.2 cm^2^, showing a mottled and smooth purple‐pink serosa. Histology showed adipose infiltration of the appendiceal submucosa, characteristic of lipomatosis (Figure 2). No neutrophilic infiltration into the muscularis propria or other inflammatory changes were seen to suggest acute appendicitis.

**Figure 2:**
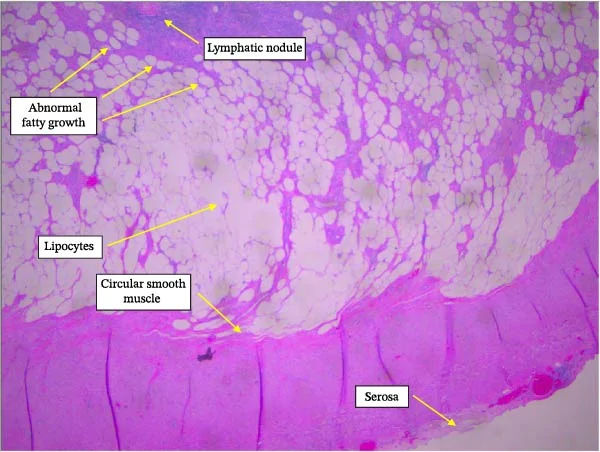
Histological report of appendiceal lipomatosis from the patient. The lipocytes can be seen infiltrating into the lymphatic nodule and the lumen of the appendix.

## 3. Discussion

Intestinal lipomatosis already represents a rare clinical entity with varying presentation; isolated appendiceal lipomatosis is exceptionally rare with only a handful of previously documented cases, the first of which was reported in 1956 by Antoci [5]. Clinically, it presents like acute appendicitis, with symptoms of RLQ pain and tenderness and occasionally systemic symptoms such as fever. Nevertheless, there are important differences to help distinguish between the two diseases. The patient described here only demonstrated RLQ pain. He lacked rebound tenderness and leukocytosis, which are commonly seen in acute appendicitis. Moreover, appendicitis normally has an acute presentation, whereas this patient was symptomatic for over a month. Leukocytosis was not observed in any of the reported cases that were available for review, which is typically a key distinguishing feature of acute appendicitis.

Table 1 highlights key information from our case as well as the other easily accessible documented cases of appendiceal lipomatosis. Compared with our case, the published literature on appendiceal lipomatosis demonstrates both shared features and notable variability among reported cases. Divyalakshmi and Banu [6] reviewed a case where a 65‐year‐old female presented with complaints of intermittent RLQ discomfort. There were no abnormalities on laboratory findings, and CT imaging demonstrated a thickened appendix with periappendiceal fat stranding. The patient proceeded for laparoscopic appendectomy, with final pathology demonstrating acute appendicitis with submucosal lipomatosis. Another case report, Sanches et al. [7] looked at a 34‐year‐old female in her second trimester of pregnancy who presented with 3 weeks of abdominal discomfort with a grossly unremarkable laboratory workup. Ultrasound demonstrated appendiceal thickening without surrounding inflammation, and further imaging with MRI showed diffuse hypersignal of the appendix in the in‐phase and loss of signal in the out‐of‐phase, demonstrating extensive fat in the intracellular compartment consistent with appendiceal lipomatosis. Compared to our study and the previously mentioned study, this patient was discharged home with pain management and close follow‐up without recurrence of symptoms. Last, Shanmugarajah et al. [8] reviewed a 16‐year‐old boy with 3 days of RLQ abdominal pain. Workup demonstrated elevation in C‐reactive protein to 56 mg/L, and the patient underwent laparoscopic appendectomy, with minimal inflammation of the appendix noted intraoperatively. The specimen was sent for pathology, demonstrating fatty tissue within the submucosa, again consistent with a diagnosis of appendiceal lipomatosis.

**Table 1:** Summary of available cases of appendiceal lipomatosis.

Patient	Age/sex	Sign/symptoms	Duration of symptoms	Leukocytosis	CRP	Treatment
This report	66	RLQ pain	1 month	None	Elevated	Appendectomy
Divyalakshmi and Banu [6]	65	RLQ pain and tenderness	Not described	None	Normal	Appendectomy
Sanches et al. [7]	34	RLQ pain	3 weeks	None	Normal	Analgesia
Shanmugarajah et al. [8]	16	RLQ pain and tenderness, fevers	3 days	None	Elevated	Appendectomy

Worldwide, the leading cause of acute abdomen remains acute appendicitis, with a prevalence of 7%–12% [9]. However, it is important to recognize other possible causes of appendix disorders as around 13% (95% CI 12%–14%) of suspected cases are not acute appendicitis [10]. The rarity of appendiceal lipomatosis presents significant challenges due to the lack of information on treatment options and decreased recognition among healthcare professionals. The primary method of treating and diagnosing appendiceal lipomatosis is by performing a laparoscopic appendectomy and then by histopathology examining the tissue for fatty infiltration. Surgery had resolved all associated symptoms in our patient as well as in the above‐mentioned patients who had undergone surgical appendectomy. The removal of this patient’s appendix was the standard of care based on a dilated appendix of 1 cm as seen on CT imaging.

## 4. Conclusion

This case highlights the importance of growing clinical studies on this disease process. Due to its rarity and similarity in presentation to acute appendicitis, cases of appendiceal lipomatosis are likely underreported and would only be confirmed after resulting pathology. Potential risk factors for this disease include obesity, insulin resistance, advanced age, and genetic predisposition. This case contributes to the limited body of literature on appendiceal lipomatosis and intestinal lipomatosis as a whole and highlights key differences that help to differentiate this disease process from acute appendicitis.

## Funding

No funding was received for this manuscript.

## Ethics Statement

Institutional Review Board approval was not required for this single‐anonymized case report.

## Consent

Written informed consent was obtained from the patient for the publication of this case report and its contained information. Data were deidentified, and the manuscript was prepared per CARE case report guidelines [11].

## Conflicts of Interest

The authors declare no conflicts of interest.

## Data Availability

Data sharing is not applicable to this article as no datasets were generated or analyzed during the current study.
